# The relationship between mindfulness and athletes’ mental skills may be explained by emotion regulation and self-regulation

**DOI:** 10.1186/s13102-024-00863-z

**Published:** 2024-03-19

**Authors:** Aleksandra M. Rogowska, Rafał Tataruch

**Affiliations:** 1 Institute of Psychology, Department of Social Sciences, University of Opole, Opole, Poland; 2 Faculty of Physical Education and Physiotherapy, Opole University of Technology, Opole, Poland

**Keywords:** Elite athletes, Interoceptive awareness, Speed skating, Sports success, State mindfulness for physical activity

## Abstract

**Background:**

Although numerous psychological determinants of sports success have been identified in the scientific literature, research on the contribution of mindfulness and interoceptive awareness to sports achievements remains limited. This study investigates the relationship between self-reported mental skills determining sports success (i.e., flow state, attention, technique, sensitivity to error, commitment, and achievement), state mindfulness for physical activity (of the mind and the body), and interoceptive awareness (including scales of noticing, not distracting, not worrying, attention regulation, emotional awareness, self-regulation, body listening, and trusting).

**Methods:**

A cross-sectional online survey was conducted on a sample of elite athletes in speed skating (*n* = 54) and university students of physical education (*n* = 102) representing various sports disciplines and competitive levels. The Sports Success Scale (SSS), the State Mindfulness Scale for Physical Activity (SMS-PA), and the Multidimensional Assessment of Interoceptive Awareness (MAIA-2) were used to assess psychological determinants of athletic achievements, state mindfulness, and interoceptive sensitivity, respectively.

**Results:**

Our findings indicate some small-to-moderate differences in particular dimensions of psychological traits related to sports success, mindfulness, and interoceptive awareness between athletes of different genders, groups, and competitive levels. A chain mediation model showed that the relationship between body mindfulness and psychological variables determining sports success is fully explained by two dimensions of interoception: self-regulation and attention regulation.

**Conclusions:**

Cultivating the mindfulness state of the body can improve self-regulation and attention regulation, which in turn may increase the mental skills required for successful sports participation. Therefore, mental training should focus primarily on body mindfulness, attention regulation, and self-regulation to improve the mental skills responsible for athletes’ sports achievements. In addition, individual differences in athletes’ gender, sports discipline, and level of sports competition should be considered during mental training.

## Background

Sports performance refers to an individual’s or team’s proficiency in competitive sports and athletic pursuits. Sports success results from the interplay of various internal and external factors that influence the development of sports skills and lead to optimal performance levels during long-term sports training. It is a multifaceted concept involving various physical, mental, and strategic factors that contribute to sports success [1–4]. Factors affecting sports performance include physical abilities (e.g., strength or speed), technical skills (e.g., technical and tactical), mental skills (e.g., concentration, confidence, anxiety control, and stress management), and achievement motivation (e.g., task and ego involvement, and mastery orientation) [1–4]. The present study will examine associations between mental skills and successful participation in sports.

The Sports Success Scale (SSS), a multidimensional measurement tool for crucial factors determining sports achievements in the psychological field, was developed by Mousavi and Vaez Mousavi [5] through an extensive review of the sports science literature and expert opinions. Factor analysis of the SSS revealed five psychological factors of high sports performance: flow state (an optimal level of arousal and mental state of maximum efficiency), attention (the ability to focus attention on a limited range of stimuli or events), technique (an appropriate practice that improves speed, technique, and tactical principles; advanced level of sports skills, accuracy, and coordination in performance; and the ability to repeat the action), sensitivity to error (ability to identify and correct errors during a performance), commitment (a sense of dependence and belonging to specific sport activity, perseverance, and stability in continuing this behavior), and achievement motivation (the need to master difficult tasks, strive for perfection, overcome difficulties and obstacles, be better than others, and a proud sense of success).

Another psychological factor important to sports performance is mindfulness. Research indicates mindfulness can enhance physical activity and sports performance [6–9]. Mindfulness is a mental state achieved by focusing on the present moment and accepting one’s feelings, thoughts, and bodily sensations without judgment or evaluation [10, 11]. It encourages the recognition of physical sensations and their relation to emotional states, increases self-awareness and concentration abilities, and improves athletes’ attention and emotional regulation. Furthermore, mindfulness practice has been found to reduce stress and anxiety and improve overall well-being [7, 12–14]. Mindfulness emphasizes the connection between the mind and body, increasing interoceptive awareness – the ability to perceive internal bodily sensations, such as hunger, thirst, temperature, heart rate, breathing patterns, muscle tension, fatigue, and overall physical discomfort [15–23]. Interoceptive awareness can be assessed using behavioral observations during stressful situations, interoceptive accuracy tasks (e.g., monitoring internal bodily sensations, such as counting heartbeats or detecting changes in breathing rate), psychophysiological assessments (of heart rate variability, skin conductance, or respiratory rate), experimental techniques, clinical interviews, and self-report questionnaires [24–35]. The Multidimensional Assessment of Interoceptive Awareness (MAIA-2) is a self-report tool designed to assess interoceptive awareness on eight dimensions: noticing (the awareness of internal bodily sensations, such as heartbeat, breathing, and muscle tension), not-distracting (the ability to stay focused on bodily sensations without being easily distracted by external stimuli), not-worrying (the tendency to worry or become anxious about bodily sensations), attention regulation (the ability to regulate attention towards bodily sensations, including shifting attention away from them when necessary), emotional awareness (the awareness of how bodily sensations correlate with emotional states, such as recognizing how one’s heart rate increases during moments of stress), self-regulation (the ability to regulate emotions and bodily sensations effectively), body listening (the ability to listen to and interpret bodily sensations as meaningful information), and trusting (the extent to which individuals trust their bodily sensations as accurate indicators of their internal state) [36].

Interoceptive awareness and mindfulness are interconnected concepts that involve paying attention to internal bodily sensations and the present moment [37, 38]. Both phenomena, mindfulness, and interoceptive awareness, involve self-reflection on the body and promote awareness of bodily sensations. While mindfulness does not differentiate between attention directed towards exteroception, interoception, or thoughts, interoceptive awareness specifically focuses on somatic experiences, but does not differentiate between different attention styles towards internal stimuli, whether mindful or anxiety-driven [37]. Developing interoceptive awareness can enhance mindfulness practice, while mindfulness can improve awareness of bodily sensations and emotions, leading to improved well-being and emotional regulation [15–17, 19–23, 30, 37–42]. To cultivate interoceptive awareness, athletes should engage in mindfulness practices, such as body scans, meditation, and yoga [43]. These practices can help individuals become more in tune with their bodily sensations and can be integrated into their training routines.

Interoception has recently gained recognition in physical activity and sports performance research as a means of improving physical and mental resilience (understood as a cognitive process focused on coping with difficult events and situations and recovering from them) by monitoring one’s inner bodily sensations and making appropriate adjustments [43–48]. It plays a crucial role in shaping athletes’ emotional experiences, decision-making processes, and well-being [43, 47, 49, 50]. Developing interoceptive awareness can enhance an athlete’s body confidence, resulting in better self-esteem and more positive body image, ultimately contributing to improved performance [50, 51]. Interoceptive awareness increases self-regulation and enables athletes to better manage their attention and emotions during competitions [50]. By being attuned to their physiological responses, such as increased heart rate and muscle tension, athletes can recognize signs of anxiety or stress and employ strategies, such as deep breathing or relaxation techniques, to remain calm and focus on high-pressure competitive situations [51]. Furthermore, athletes with a strong interoceptive awareness can better gauge their pain threshold and identify early signs of fatigue, overtraining, injury, or overuse, thereby preventing more severe injuries [52–57]. Lastly, athletes with strong interoceptive awareness can optimize their training load and use appropriate recovery strategies, such as proper rest, sleep, or nutrition, to ensure they are adequately prepared for their next training session or competition [43]. Overall, interoception has the potential to enhance athlete performance, well-being, and resilience significantly.

### The current study

Although extensive research has been conducted on athletic success for several decades, the interplay between psychological factors that significantly contribute to becoming a successful or elite athlete remains largely unknown. This lack of knowledge can have significant implications for the inappropriate selection process of various sports disciplines and the insufficient development of sports talent. Specifically, there is limited understanding of the configuration of mental skills crucial for successful participation in sports activities. In this study, we aimed to examine the complexity of perceived psychological traits, abilities and competencies, including multiple components of mental skills determining sports success (i.e., flow state, attention, technique, sensitivity to error, commitment, and achievement), as well as two multidimensional interrelated variables: interoceptive awareness (including such scales as noticing, non-distracting, not-worrying, attention regulation, emotional awareness, self-regulation, body listening, and trusting subscales) and state mindfulness in physical activity (comprising state mindfulness of the mind and body).

Elite athletes in speed skating (EASS) will be compared with university students in physical education (USPA). We will explore whether the EASS demonstrates specific self-reported psychological skills than the USPA sample. Additionally, we will examine gender (women vs. men) and competitive level (international vs. national or lower) differences. Another objective of this study is to identify the most critical psychological variables for sports achievement. Therefore, we will explore the associations between the mental dimensions of sports success, state mindfulness for physical activity, and interoceptive awareness. We are interested in the extent to which self-reported state mindfulness and interoception can explain the variance in mental athletic skills. To the best of our knowledge, these analyses have never been performed previously. Therefore, the present study has exploratory characteristics, and we do not assume any direct hypotheses.

## Materials and methods

### Participants and procedure

Initially, an *a priori* power analysis was conducted using G*Power 3.1 software [58] to determine the appropriate sample size for the study. It was determined that 51 participants per group were necessary for an independent sample Student’s *t*-test to detect an effect size of Cohen’s *d* = 0.50 with 80% power (α = 0.05). For correlation analysis, a minimal sample size of 84 people was expected, taking into account an effect size of ρ = 0.30 and 80% power (α = 0.05, two-tailed). To demonstrate an effect size of ƒ2 = 0.15 with 80% power (α = 0.05) for a linear multiple regression model with 13 predictors (when *R*^2^ increases), a minimum of 118 participants was required. Since 156 people participated in the present study, the sample size is adequate, and the post-hoc analysis showed a power of 0.91 for the *t*-test, 0.99 for the Spearman’s correlation, and 0.89 for the linear regression test.

Following approval from the IRB, recruitment of participants commenced. The cross-sectional online study was conducted in Poland between 3 August and 30 November, 2020. The survey, which included informed consent and standardized psychological questionnaires, was disseminated via e-mail to elite athletes (all members of the Polish Speed Skating Association) and physical education students from the FPEP at one university. The criterion for inclusion in both the EASS and USPA samples was a minimum age of 16 years old.

A total of 156 athletes participated in the study, including 54 EASS (25 women) and 102 USPA (40 women). The participants ranged in age from 16 to 34 years (*M* = 21.57, *SD* = 3.58), with 41.67% females. The EASS group included athletes from short-track (*n* = 15), sprint on long-track (*n* = 15), intermediate runs, and all-around-event on long-track (*n* = 23), and their average training experience was nine years (*M* = 9.11, *SD* = 4.36), ranging from 1 to 22 years. The group was divided into Junior (*n* = 28), Youth (*n* = 11), and Senior (*n* = 15) categories, with 13 individuals representing the Master International level, 18 at the Master class, 14 in the First Sports class, and 9 in the Second class. Among EASS, 17 participants were at the national competitive level, while 37 were at the international level. The USPA sample consisted of 69 undergraduates (Bachelor’s degree) and 35 graduates (Master of Science degree) in their first (*n* = 9), second (*n* = 79), and third (*n* = 15) year of study. The USPA group represented various sports disciplines, including athletics (*n* = 9), badminton (*n* = 2), basketball (*n* = 8), bodybuilding (*n* = 4), combat sports and martial arts (*n* = 8), cross-fit (*n* = 2), cycling (*n* = 1), dancing (*n* = 4), fitness (*n* = 7), football (*n* = 31), gymnastics (*n* = 2), handball (*n* = 8), swimming (*n* = 4), table tennis (*n* = 2), and volleyball (*n* = 10). Participants competed at various levels, including local or recreational (*n* = 22), regional (*n* = 34), national (*n* = 29), and international (*n* = 17).

### Measures

The survey consisted of a demographic part (including age, gender, sports discipline, training experience, sports class, and competition level) and three standardized questionnaires measuring mental skills, mindfulness, and interceptive awareness: the Sports Success Scale (SSS), the State Mindfulness Scale for Physical Activity (SMS-PA) and the Multidimensional Assessment of Interoceptive Awareness, version 2 (MAIA-2), respectively. All questionnaires were previously developed in English and validated (see references below). For this study, we translated all questionnaires from English into Polish by a bilingual specialist in sports psychology, and vice versa from Polish into English by a Polish specialist in English philology. Then, in a discussion, both experts agreed on the final Polish version of the items in the questionnaires.

#### Mental skills

The Sports Success Scale (SSS) is a self-report instrument designed to assess crucial psychological dimensions of high achievement in sports, as described by Mousavi and Vaez Mousavi [5]. The SSS comprises 29 items across six scales: Flow state (5 items), Attention (5 items), Technique (4 items), Sensitivity to error (5 items), Commitment (5 items), and Achievement (5 items). Participants rate their compliance with each item on a 6-point scale (1 = *Strongly disagree* to 6 = *Strongly agree*), with the total SSS score ranging from 29 to 174 (a higher score indicating a better personal predisposition for sports achievements). The internal consistency of the SSS, as assessed by Cronbach’s alpha, was 0.89 in the present study, with scale-specific alpha coefficients ranging from 0.61 to 0.73 for Flow state, Attention, Technique, Sensitivity to error, Commitment, and Achievement, respectively.

#### Mindfulness

The State Mindfulness Scale for Physical Activity (SMS-PA) was created by Cox et al. [59] to evaluate physical and mental mindfulness states, as well as the fundamental characteristics of mindfulness, such as attention, awareness, and openness. The SMS-PA consists of 12 items across two scales: State mindfulness of the mind (SMM, 6 items) and State mindfulness of the body (SMB, 6 items). Participants rate their level of mindfulness on a scale from 0 to 4 (0 = *Not at all* to 4 = *Very much*), with higher scores indicating greater mindfulness. The Cronbach’s alpha coefficients for the SMM and SMB scales were 0.84 and 0.89 in this study, respectively.

#### Interoceptive awareness

The Multidimensional Assessment of Interoceptive Awareness, Version 2 (MAIA-2) is a self-report questionnaire designed to assess various aspects of interoceptive awareness [36]. It evaluates an individual’s ability to recognize and interpret bodily sensations and their awareness of these sensations on 37 items across eight scales: Noticing (NO, *n* = 4), Not distracting (NT, *n* = 6), Not worrying (NW, *n* = 5), Attention regulation (AR, *n* = 7), Emotional awareness (EA, *n* = 5), Self-regulation (SR, *n* = 4), Body listening (BL, *n* = 3), and Trusting (TR, *n* = 3). Participants are asked to rate their agreement or frequency of experiencing these sensations on a 6-point Likert scale (from 0 = *Never* to 5 = *Always*). High scores indicate a high interoceptive sensibility. The internal consistency of the particular MAIA-2 scales in the present study is 0.66, 0.68, 0.75, 0.83, 0.79, 0.78, 0.73, and 0.85 for Noticing, Not distracting, Not worrying, Attention regulation, Emotional awareness, Self-regulation, Body listening, and Trusting scales, respectively.

### Statistical analysis

Descriptive statistical analyses were initially conducted to assess the normality assumption for parametric tests. As not all variables met the normality assumption (assessed using the Shapiro-Wilk test), we performed a non-parametric Mann-Whitney *U*-test to examine differences in SSS, SMS-PA, and MAIA-2 scales between genders (Women, Men), spots groups (EASS and USPA), and competitive levels (International, National or lower). Rank biserial correlation (RBC) was used to assess the effect size for *U*-test. Spearman’s correlation was conducted to assess relationships between all scales of SSS, SMS-PA, and MAIA-2. Hierarchical linear multiple regression analysis was then performed for the total SSS as a dependent variable, as well as gender, group, competitive level, and all scales of the SMS-PA and MAIA-2 questionnaires. All assumptions for linear regression were met, including multicollinearity (variance inflation factor VIF < 3, ranging between 1.19 and 2.35 in the present study; tolerance > 0.25, ranging between 0.43 and 0.84), multivariate normality (Shapiro-Wilk statistic = 0.99, *p* = 0.397), autocorrelation (Durbin-Watson statistic = 1.91, *p* = 0.484), and heteroscedasticity (Breush-Pagan statistic = 18.86, *p* = 0.127). The statistical analyses were performed using JAMOVI version 2.3.28 software for Windows.

## Results

### Intergroup differences in mental skills, mindfulness, and interoceptive awareness

The intergroup differences in mental skills, state mindfulness for physical activity, and interoceptive awareness scales were examined using the Mann-Whitney *U*-test. The results of the gender comparison showed that women scored significantly higher than men in commitment and emotional awareness. In contrast, men outperformed women in technique, not worrying, and trusting scales, although the effect size for these differences was small (Table 1). The USPA sample scored higher than the EASS group in state mindfulness for physical activity for both the mind and the body, with a moderate effect size. At the same time, EASS showed higher commitment than USPA, with a small effect size (Table 2). Athletes at the highest international competitive level (independent of sports discipline) scored higher in technique (SSS) and not worrying (MAIA-2) than those representing the national or lower competitive levels (regional, local, or recreational), but the effect size was small (Table 3). On the other hand, athletes at the international competitive level demonstrated lower scores in state mindfulness for physical activity for both the mind and the body, and the Not distracting scale of the MAIA-2, compared to those at the national or lower levels of competitions, with a small effect size.

**Table 1:** Gender differences in mental skills, mindfulness state, and interoceptive awareness

Variable	Scales	Women (*n* = 65)		Men (*n* = 91)		*U*	*p*	RBC
		*M*	*SD*	*M*	*SD*			
**Mental skills**	Flow state	21.00	3.50	21.73	4.03	2611.0	0.212	-0.117
	Attention	21.77	3.24	22.32	4.34	2652.5	0.272	-0.103
	Technique	16.05	3.35	17.69	3.43	2131.0	0.003	-0.279
	Sensitivity to error	21.12	2.37	21.78	3.76	2653.0	0.272	-0.103
	Commitment	26.02	2.79	24.48	4.21	3572.5	0.026	0.208
	Achievement	20.25	3.66	20.53	4.73	2722.5	0.398	-0.079
	Total sport success	126.20	12.17	128.53	19.63	2618.0	0.223	-0.115
**State** **mindfulness**	Mind mindfulness	20.31	4.59	19.85	4.69	3117.0	0.566	0.054
	Body mindfulness	20.42	5.85	20.63	5.83	2841.0	0.676	-0.039
**Interoceptive awareness**	Noticing	3.48	0.84	3.22	0.95	3484.0	0.058	0.178
	Not distracting	1.86	0.69	1.92	0.70	2828.5	0.643	-0.044
	Not worrying	2.20	0.98	2.69	0.72	1956.0	< 0.001	-0.339
	Attention regulation	3.17	0.76	3.29	0.79	2663.5	0.290	-0.099
	Emotional awareness	3.78	0.85	3.33	0.91	3838.0	0.002	0.298
	Self regulation	2.67	1.04	3.03	0.85	2457.5	0.071	-0.169
	Body listening	2.96	1.04	3.08	0.95	2774.5	0.509	-0.062
	Trusting	3.28	1.01	3.80	0.86	2079.5	0.001	-0.297

Note. For the Mann-Whitney *U*-test, effect size is given by the rank biserial correlation (RBC).

**Table 2:** Differences in mental skills, mindfulness state, and interoceptive awareness scales between university students of physical education (USPA) and elite athletes of speed skating (EASS)

Variable	Scales	USPA (*n* = 102)		EASS (*n* = 54)		*U*	*p*	BRC
		*M*	*SD*	*M*	*SD*			
**Mental skills**	Flow state	21.75	3.56	20.82	4.25	3067.0	0.242	0.114
	Attention	21.74	3.97	22.76	3.78	2268.5	0.070	-0.176
	Technique	17.04	3.40	16.94	3.67	2753.5	1.000	-22.16
	Sensitivity to error	21.44	3.38	21.63	3.06	2597.0	0.558	-0.057
	Commitment	24.43	3.92	26.43	3.04	1826.5	< 0.001	-0.337
	Achievement	20.47	4.22	20.30	4.51	2708.5	0.866	-0.017
	Total sport success	126.86	17.80	128.87	15.20	2592.5	0.549	-0.059
**State** **mindfulness**	Mind mindfulness	21.81	3.82	16.70	4.22	4629.0	< 0.001	0.681
	Body mindfulness	22.97	4.77	15.94	4.77	4736.5	< 0.001	0.720
**Interoceptive awareness**	Noticing	3.23	0.90	3.51	0.92	2327.5	0.111	-0.155
	Not distracting	1.95	0.65	1.79	0.76	3098.0	0.199	0.125
	Not worrying	2.45	0.72	2.56	1.10	2631.0	0.647	-0.045
	Attention regulation	3.21	0.70	3.28	0.92	2561.0	0.472	-0.070
	Emotional awareness	3.45	0.81	3.63	1.07	2359.0	0.140	-0.143
	Self regulation	2.99	0.86	2.67	1.07	3203.5	0.093	0.163
	Body listening	3.09	0.87	2.91	1.17	3000.5	0.356	0.090
	Trusting	3.55	0.94	3.64	1.00	2536.0	0.413	-0.079

Note. For the Mann-Whitney *U*-test, effect size is given by the rank biserial correlation (RBC).

**Table 3:** Differences in mental skills, mindfulness state, and interoceptive awareness scales between athletes at international and other competitive levels

Variable	Scales	National or lower (*n* = 102)		International (*n* = 54)		*U*	*p*	BRC
		*M*	*SD*	*M*	*SD*			
**Mentals skills**	Flow state	21.33	3.96	21.59	3.58	2628.0	0.639	-0.046
	Attention	21.81	4.01	22.61	3.72	2358.5	0.140	-0.144
	Technique	16.50	3.57	17.96	3.12	2108.0	0.016	-0.235
	Sensitivity to error	21.28	3.31	21.94	3.16	2437.5	0.237	-0.115
	Commitment	24.78	3.87	25.78	3.45	2310.5	0.097	-0.161
	Achievement	20.46	3.98	20.32	4.90	2712.0	0.877	-0.015
	Total sport success	126.16	17.64	130.20	15.28	2351.0	0.134	-0.146
**State mindfulness**	Mind mindfulness	20.80	4.52	18.61	4.57	3519.0	0.004	0.278
	Body mindfulness	21.48	5.53	18.76	5.98	3512.0	0.005	0.275
**Interoceptive awareness**	Noticing	3.31	0.87	3.36	1.00	2628.0	0.639	-0.046
	Not distracting	1.96	0.67	1.78	0.73	3238.0	0.071	0.176
	Not worrying	2.32	0.77	2.80	0.97	1916.5	0.002	-0.304
	Attention regulation	3.16	0.75	3.38	0.83	2338.0	0.121	-0.151
	Emotional awareness	3.53	0.85	3.49	1.02	2753.0	0.999	-40.310
	Self regulation	2.84	0.88	2.96	1.08	2445.0	0.248	-0.112
	Body listening	3.05	0.93	2.98	1.08	2833.5	0.767	0.029
	Trusting	3.53	0.90	3.68	1.05	2357.0	0.136	-0.144

Note. For the Mann-Whitney *U*-test, effect size is given by the rank biserial correlation (RBC).

### Associations between mental skills, mindfulness, and interoceptive awareness

Initially, Spearman’s correlation analyses were conducted to investigate how state mindfulness for physical activity and interoceptive awareness are related to mental skills responsible for sports success (Table 4). Mind mindfulness was unrelated to the SSS scales. Body mindfulness was positively related to FS, TE, and the total SSS score. Most of the interoceptive awareness scales were positively associated with the total SSS score. However, ND was negatively related to the SSS, while EA was unrelated. Considering associations between particular subscales of the SSS and dimensions of interoceptive awareness, FS was positively related to NO, NW, AR, SR, BL, and TR, and negatively to ND. The AT and SE scales of the SSS were positively correlated with the NO, AR, BL, and TR scales of interoceptive awareness. The technique scale (TE) was found to correlate positively with NW, AR, and TR, while negatively correlated with ND. Commitment (CM) positively correlated with most scales of interoception (excluding SR and NW), while negatively associated with ND. Finally, AC has a negative correlation with ND and a positive correlation with AR. All significant correlations were small in magnitude, ranging between 0.16 and 0.38.

**Table 4:** Spearman’s correlations between the Sports Success Scale (SSS), State Mindfulness for Physical Activity (SMS-PA), and Multidimensional Assessment of Interoceptive Awareness (MAIA-2)

Variables		Sports Success Scales (SSS)						
		FS	AT	TE	SE	CM	AC	SSS
**State mindfulness for PA**								
	Mind mindfulness	0.05	-0.08	0.05	-0.01	-0.13	0.08	0.02
	Body mindfulness	0.29***	0.09	0.20*	0.13	-0.04	0.10	0.22**
**Interoceptive awareness**								
	Noticing (NO)	0.23**	0.21**	-0.01	0.28***	0.27***	0.05	0.24**
	Not distracting (ND)	-0.18*	-0.06	-0.18*	-0.14	-0.18*	-0.17*	-0.21*
	Not worrying (NW)	0.18*	0.15	0.16*	0.07	-0.04	0.13	0.18*
	Attention regulation (AR)	0.38***	0.30***	0.20*	0.29***	0.24**	0.19*	0.38***
	Emotional awareness (EA)	0.15	0.10	-0.03	0.13	0.35***	0.03	0.14
	Self-regulation (SR)	0.26**	0.12	0.08	0.11	0.10	0.07	0.18*
	Body listening (BL)	0.36***	0.25**	0.15	0.24**	0.27***	0.16	0.34***
	Trusting (TR)	0.29***	0.21**	0.22**	0.24**	0.18*	0.14	0.28***

Note. PA = physical activity, FS = Flow state, AT = Attention, TE = Technique, SE = Sensitivity to error, CM = Commitment, AC = Achievement, SSS = Total score of the Sports Success Scale. *N* = 163. *p < 0.05, **p < 0.01, ***p < 0.001

A hierarchical linear multiple regression analysis was conducted to determine the extent to which each set of variables (mindfulness, interoceptive awareness) contributes to the unique variance in mental skills determining sports success among athletes (Table 5). In the first step of the analysis, bi-categorical variables, including gender (coded Women = 0, Men = 1), group (USPA = 0, EASS = 1), and competitive level (National or lower = 0, International = 1), were entered into the model. The resulting regression model was found insignificant, with an *R*-value of 0.13, *R*^2^ of 0.02, and an *F* value of 0.82 (*p* > 0.05), explaining only 2% of the variance in psychological variables determining sports success. However, when two scales of state mindfulness for physical activity were added to the model in the second step, the regression results significantly improved, with an *R*-value of 0.29, *R*^2^ of 0.08, and an *F* value of 2.63 (*p* < 0.05), explaining 8% of the variance in mental skills related to sport success. Body mindfulness was found to be a significant positive predictor of the total SSS score (β = 0.30, *p* < 0.01). In the third step of the regression analysis, all scales of the interoceptive awareness were included in the model, resulting in a significant increase in explained variance to 26%, with an *R*-value of 0.51, *R*^2^ of 0.26, and an *F* value of 3.79 (*p* < 0.001). However, only two scales of the MAIA-2 (AR and SR) were found to be significant predictors of mental skills (β = 0.28, *p* < 0.01 and β = -0.22, *p* < 0.05, respectively), while the state mindfulness of the body was no longer significant. The present results suggest that both self-regulation and attention regulation may play a mediating role in the association between body mindfulness and psychological variables determining sports performance.

**Table 5:** Linear multiple regression for the total score of the Sport Success Scale (*N* = 163)

			95% CI									
Predictor	b	SE b	Lower	Upper	t	p	β	ΔR²	F	df_1_	df_2_	p
Intercept	125.80	2.12	121.60	130.00	59.24	< 0.001		0.02	0.82	3	149	0.483
Gender (Men)	2.40	2.44	-2.42	7.22	0.98	0.327	0.16					
Group (EASS)	-0.19	3.01	-6.15	5.76	-0.06	0.950	-0.01					
Level (International)	3.11	2.98	-2.78	9.01	1.04	0.298	0.21					
Intercept	105.58	7.77	90.23	120.94	13.59	< 0.001		0.07	5.28	2	147	0.006
Gender (Men)	2.78	2.38	-1.94	7.49	1.16	0.246	0.19					
Group (EASS)	5.85	3.53	-1.13	12.82	1.66	0.100	0.40					
Level (International)	2.43	2.91	-3.32	8.17	0.84	0.405	0.16					
Mind mindfulness	0.10	0.34	-0.57	0.77	0.30	0.768	0.03					
Body mindfulness	0.78	0.28	0.23	1.34	2.79	0.006	0.30					
Intercept	92.83	10.69	71.71	113.96	8.69	< 0.0001		0.18	4.22	8	139	< 0.001
Gender (Men)	1.48	2.53	-3.52	6.49	0.59	0.559	0.10					
Group (EASS)	-0.34	3.48	-7.23	6.54	-0.10	0.922	-0.02					
Level (International)	1.84	2.87	-3.83	7.51	0.64	0.522	0.12					
Mind mindfulness	-0.27	0.33	-0.92	0.38	-0.82	0.415	-0.08					
Body mindfulness	0.43	0.28	-0.13	0.99	1.53	0.128	0.16					
Noticing	0.97	1.52	-2.03	3.98	0.64	0.523	0.06					
Not distracting	-1.68	1.75	-5.13	1.78	-0.96	0.338	-0.08					
Not worrying	1.75	1.49	-1.19	4.70	1.18	0.241	0.10					
Attention regulation	5.38	1.77	1.89	8.87	3.05	0.003	0.28					
Emotional awareness	0.65	1.69	-2.69	4.00	0.39	0.699	0.04					
Self regulation	-3.43	1.61	-6.61	-0.26	-2.14	0.034	-0.22					
Body listening	2.94	1.58	-0.19	6.06	1.86	0.065	0.19					
Trusting	2.05	1.40	-0.72	4.82	1.47	0.145	0.13					

**Fig. 1:**
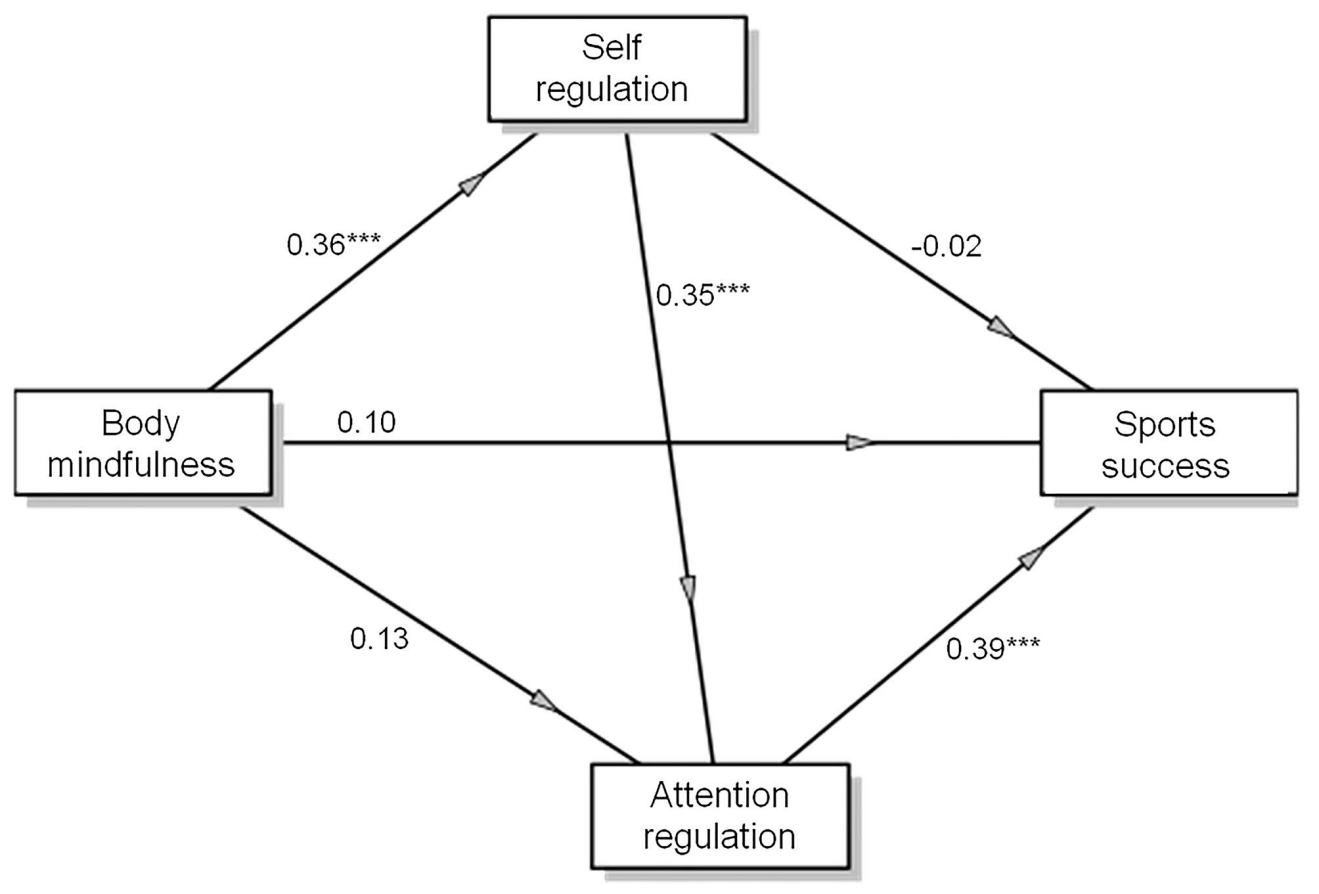
A chain mediation model of the association between the state of mindfulness of the body and sports success via self-regulation and self-attention scales of interoception. ***p < 0.001

Undertaking a GLM mediation analysis, we sought to examine the mediating role of self-regulation and attention regulation in the relationship between body mindfulness and sports success (as depicted in Table 6; Fig. 1). Our analysis revealed a chain mediating effect of body mindfulness on sports success, whereby both self-regulation and attention regulation scales of interoceptive awareness were involved. The chain mediation model of regulation-based interoceptive abilities fully mediated the association between body mindfulness (as measured by the SMS-PA) and sports success (as assessed by the SSS). Specifically, self-regulation and attention regulation served as complete mediators in the relationship between body mindfulness and sports success (as illustrated in Fig. 1).

**Table 6:** The mediating effect of Attention regulation (AR) for the association between mindfulness of the body (MB) and total score of the Sports Success Scale (SSS)

				BC 95% CI				
Type	Effect	b	SE b	Lower	Upper	β	z	p
**Indirect**	MB ⇒ SR ⇒ SSS	-0.02	0.08	-0.17	0.14	-0.01	-0.24	0.809
	MB ⇒ AR ⇒ SSS	0.13	0.11	-0.06	0.39	0.05	1.22	0.222
	MB ⇒ SR ⇒ AR ⇒ SSS	0.13	0.06	0.05	0.29	0.05	2.13	0.033
**Component**	MB ⇒ SR	0.06	0.01	0.03	0.09	0.36	4.27	< 0.001
	SR ⇒ SSS	-0.30	1.21	-2.45	2.24	-0.02	-0.25	0.804
	MB ⇒ AR	0.02	0.01	-0.01	0.04	0.13	1.34	0.181
	AR ⇒ SSS	7.50	1.69	4.18	10.77	0.39	4.44	< 0.001
	SR ⇒ AR	0.29	0.08	0.15	0.45	0.35	3.77	< 0.001
**Direct**	MB ⇒ SSS	0.25	0.24	-0.26	0.70	0.10	1.05	0.292
**Total**	MB ⇒ SSS	0.50	0.21	0.09	0.91	0.19	2.38	0.017

Note. Confidence intervals computed with Bias Corrected (BC) bootstrap method using 1000 replications. Betas are completely standardized effect sizes. CI = confidence interval

## Discussion

The primary objective of this study was to investigate the most critical psychological factors that impact both objective measures of sports achievement, such as representing a national speed skating team and competing at international levels, and mental skills determining sports success. Our findings indicate that gender has a minimal relationship with the mental skills of sports success and interoceptive awareness, and no association with state mindfulness for physical activity. Women are more engaged in sports activities and are more emotionally aware of their interoceptive signals than men. However, men have improved women’s sports techniques and are better at not worrying about and trusting their bodies. Our results align with previous studies [25, 60–63]. The observation that women often score higher than men in emotional awareness can be attributed to a combination of biological, social, and cultural factors [61–63]. Biological differences concern brain structure and function related to emotional processing [60]. The size and connectivity of brain regions involved in emotional regulation and empathy, such as the amygdala and prefrontal cortex, promote better emotional processing and higher emotional intelligence in women than men. Awareness of emotion-related body states depends on both internal physiological signals and external situational signals [61, 63]. Women differ from men in that they prefer to use different cues when defining their emotional state, namely, in a way consistent with cognitive appraisals. Gender differences may also result from biases in the interpretation of ambiguous internal body states, which are determined by gender-specific linguistic socialization. During the socialization process, girls may receive more encouragement to talk about their feelings, express empathy, and engage in emotionally expressive behaviors.

On the other hand, boys may be socialized to suppress emotions, leading to lower levels of emotional awareness and expression. Additionally, cultural norms and expectations regarding gender roles and expression may interact with gender-specific emotional socialization to shape attitudes toward emotions and influence emotional awareness and expression. Women may experience different psychosocial factors and distinct stressors than men, and have different coping mechanisms compared to men, which can impact their emotional awareness and regulation.

In addition, we aimed to examine whether successful athletes differ significantly from those with lower levels of sports achievement. We addressed this question by comparing elite athletes with physical activity students and athletes representing international and lower competitive levels. The sample of elite athletes in speed skating (EASS, members of the Polish National Olympic Representation) showed higher sports commitment than those representing various sports disciplines and competitive levels (university students of physical activity, USPA), but on a small effect size. On the other hand, the EASS sample scored significantly lower in both scales of state mindfulness for physical activity (of the mind and body) than the USPA group. A high competition level and pressure to win among EASS can reduce mindfulness skills, dwelling on past failures, or worrying about the future. Previous studies revealed that competitive anxiety related to high sports pressure (internal, e.g., perfectionism-related, and external pressure of other team members, coaches, family, and significant others) and uncertainty stress are negatively related to mindfulness [64–72]. In particular, mindfulness traits act as mediators and protective factors in the effects of impulsivity on anxiety among female athletes [69].

The differences between athletes competing at the international level and those at national or lower competitive levels may be attributed to superior sports techniques and the ability to remain focused amidst intense competition. It is plausible that extensive experience in competition fosters these skills. Unfortunately, athletes participating in international competitions scored lower in mind and body state mindfulness, which refers to the ability to remain present and focused during physical activity, than their counterparts at the national or lower level. Top athletes may face more challenges than lower-level athletes. Therefore, they should routinely practice mindfulness skills [73, 74]. Inadequate mindfulness skills can make individuals more susceptible to distractions, which can negatively affect concentration and sports performance [6–9].

On the other hand, mindfulness practice has been shown to enhance physical activity and sports performance. Therefore, it can be inferred that the EASS sample, as well as athletes at the highest international competitive level, would benefit from increased mindfulness training to enhance their sports performance. Indeed, research showed that systematic mindfulness practice and experiential acceptance approaches can significantly reduce competitive anxiety, rumination, experiential avoidance, and emotion regulation difficulties, and simultaneously increase positive emotions and self-efficacy, improving attentional control, mindfulness, flow, and performance in elite sports [6, 7, 75–79]. The mindfulness-based intervention also has beneficial effects on executive functions in athletes [8, 80, 81].

Interoceptive processes play a critical role in regulating physical exertion during physical activities and sports [43, 49]. Previous research has demonstrated that a co-designed exercise intervention can improve interoceptive awareness and mental health in non-athletes [44]. Additionally, a moderate-vigorous physical activity-based intervention can increase cardiac interoceptive accuracy, and individuals with a higher sports background tend to benefit more than those who are physically inactive [47]. Research further indicates that sprinters and distance runners outperform non-athletes in areas such as body trust, attention regulation, and self-regulation [50].

Our investigation uncovered a novel finding that body mindfulness for physical activity influences the psychological dimensions of sports success through a series of interoceptive regulatory skills, specifically self-regulation and attention regulation. A high degree of mindfulness in physical activity is a predictor of elevated self-regulation, which subsequently leads to enhanced attention regulation skills and greater mental skills in sports success among athletes. Previous research has indicated that mindfulness training can improve interoceptive awareness [22, 44, 47, 48, 82]. Thus, it is crucial to cultivate mindfulness practice alongside physical training to increase open awareness by consciously paying attention to the present moment, engaging in breath observation, and encouraging the recognition of internal physical sensations and their connection to emotional states without judgment [6–9]. Mindfulness may offer various benefits, including reduced stress, improved emotional regulation, increased enjoyment and fulfillment from physical activity, heightened self-awareness, concentration, and performance, which ultimately determine injury prevention, resilience, recovery, and overall well-being [7, 12–14].

Despite the clear evidence provided by this study regarding the existence of psychological factors determining athletic performance, certain limitations preclude generalization. First, the studies were cross-sectional, meaning that any cause-and-effect relationship presented in the regression and mediation analyses should be considered with caution. Future research should be longitudinally performed to examine the interplay and dynamic changes in mindfulness, interoceptive awareness, and psychological skills related to sports performance during the long-term process of sports activity and development. Although the sample size met the minimum requirements for power analysis, further research could replicate the current findings with a more representative sample for various individual and team sports. Second, all variables were self-reported, which is related to biases resulting from subjective assessments and cognitive distortions (e.g., defense mechanisms, false beliefs, such as about perfectionism, one’s abilities, or self-efficacy). It is uncertain whether the same results would be obtained if interoceptive awareness and mental skills related to sports success could be assessed using more objective measurements. Therefore, future research should use more objective psychophysiological methods to assess mindfulness and interoceptive awareness, as well as external and more objective assessments by competent judges (sports coaches, referees, and psychologists) in assessing psychological abilities. Additionally, an experimental study with mindfulness training among athletes would be more appropriate to examine the mediating effect of self-regulation and attention regulation on the association between mindfulness and the psychological dimensions of sports success. Therefore, future research should address these issues. Furthermore, different sports areas should be included in future studies to examine differences between various sports disciplines in mindfulness, interoceptive awareness, and psychological skills.

## Conclusions

The present research unveiled disparities among athletes in terms of self-reported mindfulness, interoceptive awareness, and psychological dimensions of athletic success, which were influenced by factors such as gender, level of competition, and sports discipline (being a member of a prestigious team of elite athletes). Therefore, sports psychologists and coaches should be aware of these differences and adapt training to athletes’ specific needs. Among all the psychological variables examined, body state mindfulness for physical activity, self-regulation, and attention regulation skills of interoceptive awareness were found to be the most crucial for sports success. Additionally, self-regulation and attention regulation were discovered to completely mediate the relationship between body mindfulness and sports success. These findings have significant implications for sports psychologists, who should incorporate mindfulness practices to enhance athletes’ self-regulation and attention-regulation abilities, and subsequently improve sports performance among athletes.

## Data Availability

The datasets generated for this study can be found in the Mendeley Data repository: Rogowska, A, Tataruch R. Comparison of interoceptive awareness, state mindfulness for physical activity, and sports success between elite speed skating athletes and college athletes (2023) Mendeley Data, V1. doi: 10.17632/vf5khf739w.1.
